# Trust-Aware and Energy-Efficient Federated Learning for Secure Sensor Networks at the Edge

**DOI:** 10.3390/s26082307

**Published:** 2026-04-09

**Authors:** Manuel J. C. S. Reis

**Affiliations:** Engineering Department and IEETA, University of Trás-os-Montes e Alto Douro, 5000-801 Vila Real, Portugal; mcabral@utad.pt

**Keywords:** federated learning, sensor networks, trust management, edge intelligence, energy efficiency, secure distributed learning, Internet of Things

## Abstract

The widespread adoption of large-scale sensor networks in privacy-sensitive and safety-critical applications has intensified the demand for secure, trustworthy, and energy-efficient learning mechanisms at the network edge. Federated learning has emerged as a promising paradigm for privacy preservation by enabling collaborative model training without sharing raw sensor data. However, most existing federated approaches inadequately address trust management, communication efficiency, and energy constraints, which are critical in real-world sensor-based systems. This paper proposes a trust-aware and energy-efficient federated learning framework specifically designed for secure sensor networks operating in resource-constrained edge environments. The proposed approach integrates lightweight trust metrics, trust-driven model aggregation, and adaptive communication scheduling to mitigate the impact of unreliable or malicious nodes while reducing unnecessary energy expenditure. By dynamically weighting client contributions based on trust and participation efficiency, the framework enhances robustness and learning stability under heterogeneous sensing conditions. Experimental results show that the proposed method maintains significantly higher accuracy under adversarial participation while reducing communication overhead and cumulative energy consumption. In particular, the framework improves model accuracy by up to 3.2% under heterogeneous conditions, reduces communication overhead by 28%, and decreases cumulative energy consumption by 31% compared with conventional federated learning approaches.

## 1. Introduction

The rapid proliferation of intelligent sensor networks has enabled a wide range of applications in smart cities, environmental monitoring, industrial automation, and safety-critical cyber–physical systems. These systems increasingly rely on data-driven models deployed close to the sensing infrastructure, where low latency, privacy preservation, and operational resilience are essential requirements. However, the distributed and resource-constrained nature of sensor networks introduces significant challenges related to data security, trustworthiness of participating nodes, and energy efficiency [1,2,3].

Traditional centralized learning paradigms require raw sensor data to be transmitted to a central server for processing and model training. In addition to incurring high communication overhead, this approach raises serious privacy and security concerns, particularly in applications involving sensitive or regulated data streams [4]. Federated learning (FL) has emerged as a promising alternative by enabling collaborative model training across distributed devices while keeping raw data local [5]. By exchanging model updates rather than sensor measurements, FL reduces privacy risks and alleviates bandwidth demands, making it particularly attractive for edge-based sensor systems and Internet-of-Things (IoT) deployments [6,7].

Despite these advantages, most existing federated learning frameworks implicitly assume reliable and honest participants, stable communication links, and sufficient energy resources. Such assumptions rarely hold in real-world sensor networks, where nodes may exhibit heterogeneous sensing quality, intermittent connectivity, limited battery capacity, or even adversarial behavior [8,9]. In particular, compromised or low-quality sensor nodes can degrade the global model through poisoned or noisy updates, while frequent communication rounds can rapidly deplete energy resources and limit system scalability [10].

Recent research has investigated robustness and security enhancements for federated learning, including Byzantine-resilient aggregation, anomaly detection, and secure update mechanisms [5,11,12]. While these approaches improve resilience against specific attack models, many of them focus primarily on statistical robustness or cryptographic protection, often neglecting trust dynamics and energy-aware operation at the sensor level. Trust management—understood as the continuous assessment of node reliability based on historical behavior and contribution quality—remains an underexplored yet critical dimension for secure and sustainable federated sensor systems [13,14].

In this work, we address these challenges by proposing a trust-aware and energy-efficient federated learning framework tailored for secure sensor networks operating at the edge. The proposed approach integrates lightweight trust metrics derived from model update quality and temporal consistency with a trust-driven aggregation strategy that adaptively weights client contributions. In parallel, an energy-aware communication scheduling mechanism regulates client participation to reduce unnecessary transmissions while preserving learning performance. By jointly considering trust, robustness, and energy efficiency, the proposed framework enhances resilience against unreliable or malicious sensor nodes under heterogeneous and resource-constrained conditions. Unlike prior work that treats robustness, trust, and energy efficiency as separate design objectives, this study presents an integrated framework that jointly addresses these dimensions in sensor-based federated learning systems.

The main contributions of this paper can be summarized as follows:

- We introduce a trust-aware federated learning framework specifically designed for sensor networks deployed in edge environments.
- We propose a lightweight trust metric and a trust-driven aggregation strategy to mitigate the impact of unreliable or adversarial sensor nodes.
- We incorporate energy-aware communication scheduling to reduce communication overhead and extend sensor network lifetime.
- We demonstrate, through experimental evaluation, that the proposed approach improves robustness, communication efficiency, and learning stability compared with conventional federated learning baselines.

The remainder of this paper is organized as follows. Section 2 reviews related work on secure federated learning and trust mechanisms in sensor networks. Section 3 presents the system model and threat assumptions. Section 4 details the proposed trust-aware and energy-efficient federated learning framework. Section 5 reports experimental results and discusses performance trade-offs. Finally, Section 6 concludes the paper and outlines directions for future research.

## 2. Related Work

### 2.1. Federated Learning in Sensor and Edge Networks

Federated learning (FL) has gained significant attention as a distributed learning paradigm that enables collaborative model training without requiring the exchange of raw data. Since its formal introduction, FL has been widely adopted in edge and Internet-of-Things (IoT) scenarios, where privacy preservation, bandwidth efficiency, and regulatory compliance are critical concerns [1]. In sensor networks, FL is particularly attractive due to the sensitive nature of sensed data and the increasing shift toward on-device intelligence at the network edge.

Several surveys and studies have explored the application of FL in IoT and edge computing environments, highlighting its potential to reduce communication overhead and protect data privacy while enabling scalable learning across heterogeneous devices [2,15]. However, these works also emphasize that classical FL formulations often rely on idealized assumptions, such as reliable participants, homogeneous data distributions, and stable communication conditions—assumptions that are rarely satisfied in real-world sensor networks.

In practice, sensor-based systems are characterized by heterogeneous sensing quality, intermittent connectivity, and severe resource constraints, which can significantly impact learning convergence and model performance. These challenges motivate the need for FL frameworks that are explicitly designed for sensor and edge environments, rather than directly adapting approaches originally developed for mobile devices or data centers.

### 2.2. Security and Robustness in Federated Learning

Security has emerged as a central concern in federated learning, as the distributed nature of FL exposes the learning process to a variety of adversarial threats. Unlike centralized training, FL is vulnerable to model poisoning, data poisoning, and backdoor attacks, where malicious participants intentionally manipulate local updates to corrupt the global model [4,5].

To address these threats, a significant body of work has focused on Byzantine-robust aggregation techniques, such as coordinate-wise median, trimmed mean, and Krum-based methods [5,6,11,16]. These approaches aim to tolerate a bounded number of adversarial clients by filtering or down-weighting anomalous updates during aggregation. While effective against specific attack models, Byzantine-robust methods often incur increased computational complexity and may degrade learning performance under benign but highly heterogeneous conditions.

Complementary approaches have explored secure aggregation protocols, differential privacy mechanisms, and cryptographic techniques to protect model updates during transmission and training [10,17]. These methods aim to guarantee confidentiality, integrity, and privacy of the exchanged updates; for example, through encrypted aggregation or noise-injection strategies. While such techniques significantly enhance privacy and communication security, they do not directly address the problem of unreliable or low-quality model updates originating from compromised or poorly performing sensor nodes.

Consequently, cryptographic and privacy-preserving mechanisms are largely complementary to robustness-oriented approaches. In particular, trust-aware aggregation strategies can operate jointly with secure aggregation or differential privacy techniques to improve the reliability of federated learning systems deployed in heterogeneous sensor networks.

### 2.3. Trust-Aware Federated Learning

Trust-aware federated learning has recently emerged as a promising direction to address the limitations of purely statistical or cryptographic robustness mechanisms. Trust-based approaches aim to assess the reliability of participating clients by evaluating their historical behavior, contribution quality, or consistency across training rounds, and to use this information to guide aggregation or client selection decisions.

Several studies have introduced trust models for FL in IoT and edge environments, demonstrating that trust-weighted aggregation can effectively mitigate the impact of unreliable or malicious participants [17,18,19,20]. By incorporating trust scores into the aggregation process, these methods provide a more flexible and adaptive alternative to hard outlier rejection, particularly in scenarios with gradual performance degradation or non-stationary data distributions.

Recent surveys have highlighted trust management as a key open challenge in federated edge learning, noting that trust-aware mechanisms remain underexplored compared to robustness and privacy-preserving techniques [14,15]. Moreover, many existing trust-based approaches rely on complex reputation systems or require extensive monitoring data, which may be impractical for resource-constrained sensor nodes. This gap underscores the need for lightweight trust metrics that can be seamlessly integrated into FL workflows without imposing excessive computational or communication overhead.

### 2.4. Energy-Efficient and Communication-Aware Federated Learning

Energy efficiency is a fundamental constraint in sensor networks, where nodes are often battery-powered and deployed in environments with limited access to recharging or maintenance. In federated learning, energy consumption is strongly influenced by client participation frequency, local computation costs, and communication overhead, making naïve FL implementations unsuitable for long-term sensor deployments.

To address this issue, several works have investigated energy-aware client selection and communication-efficient FL strategies. Approaches based on adaptive participation, partial model updates, or communication scheduling have demonstrated significant reductions in energy consumption while maintaining acceptable learning performance [6,8,21]. These techniques are particularly relevant for sensor networks with heterogeneous energy budgets and dynamic operating conditions.

More recent studies have emphasized the importance of jointly optimizing learning performance, communication efficiency, and resource utilization in edge-based FL systems [10]. However, energy-aware methods are often developed independently of security and trust considerations, potentially allowing malicious or low-quality nodes to exploit reduced participation constraints. This separation highlights a critical gap in the literature: the lack of integrated frameworks that simultaneously address trust, security, and energy efficiency in federated sensor networks.

### 2.5. Positioning of This Work

In contrast to existing approaches, this work proposes an integrated trust-aware and energy-efficient federated learning framework specifically tailored for secure sensor networks at the edge. By combining lightweight trust assessment, trust-driven aggregation, and adaptive communication scheduling, the proposed method addresses key limitations of prior FL, security-focused, and energy-aware solutions. This holistic perspective enables robust and scalable learning in heterogeneous and resource-constrained sensor environments, bridging an important gap in current federated learning research.

## 3. System Model and Threat Assumptions

### 3.1. System Architecture

We consider a federated learning system deployed over a distributed sensor network operating at the network edge. The system consists of a set of sensor nodes (clients) and a coordinating edge server responsible for model aggregation and orchestration. Each sensor node collects local measurements and performs on-device learning using its private data, without sharing raw sensor readings with the server or other nodes.

The learning process follows the standard federated learning paradigm. At each communication round, the edge server broadcasts the current global model to a subset of participating sensor nodes. Each selected node performs local training using its own dataset and computes a model update, which is then transmitted back to the server. The server aggregates the received updates to obtain an updated global model, which is redistributed in the next round.

Unlike conventional FL settings, the considered sensor network operates under heterogeneous conditions. Sensor nodes may differ in sensing quality, data distributions, computational capabilities, communication reliability, and available energy resources. These characteristics are explicitly considered in the system design, as they significantly influence learning stability, robustness, and long-term system sustainability.

**Figure 1:**
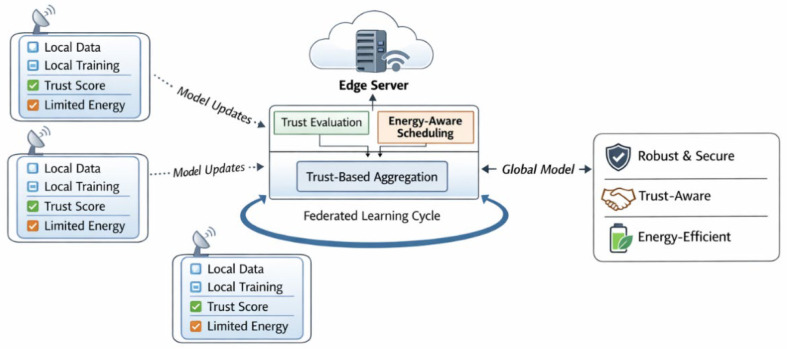
Overview of the proposed trust-aware and energy-efficient federated learning framework for secure sensor networks at the edge. Sensor nodes perform local training using private data and transmit model updates to the edge server. Trust evaluation and energy-aware participation mechanisms guide client selection and aggregation to enhance robustness, security, and efficiency.

Figure 1 illustrates the overall architecture and learning workflow of the proposed trust-aware and energy-efficient federated learning framework for secure sensor networks at the edge. The diagram highlights the interaction between distributed sensor nodes and the edge server, as well as the integration of trust assessment and energy-aware communication within the federated learning loop.

### 3.2. Data and Learning Model

Let N={1,2,…,N} denote the set of sensor nodes participating in the federated learning process. Each node i∈N holds a local dataset $D_{i}$, generated from its sensing environment. The data distributions across nodes are not assumed to be independent and identically distributed (non-IID), reflecting realistic sensor deployment scenarios.

The objective of the federated learning system is to minimize a global loss function of the form

$$\underset{w}{\min}F\left(w\right)=\sum_{i=1}^{N}p_{i}\;F_{i}\left(w\right),$$

where w denotes the global model parameters, $F_{i}\left(w\right)$ is the local loss function at node i, and $p_{i}$ represents the relative contribution weight of node i, typically proportional to the size of its local dataset.

Local training is performed for a fixed number of epochs using stochastic gradient-based optimization. Due to heterogeneity in sensing conditions and resource availability, the quality and reliability of local updates may vary significantly across nodes and over time.

### 3.3. Communication and Energy Model

Communication between sensor nodes and the edge server occurs over wireless links, which may be subject to packet loss, latency variations, and bandwidth limitations. Each communication round incurs a non-negligible energy cost associated with both local computation and model transmission.

Let $E_{i}^{\mathrm{comp}}$ and $E_{i}^{\mathrm{comm}}$ denote the energy consumption of node *i* for local computation and communication, respectively, during a given training round. The total energy expenditure per round is therefore given by

$$E_{i}=E_{i}^{\mathrm{comp}}+E_{i}^{\mathrm{comm}}.$$

To reflect realistic wireless communication conditions, communication energy consumption was modeled as distance-dependent:

$$E_{i}^{\mathrm{comm}}=P_{tx}\cdot t_{i}\cdot d_{i}^{\gamma }$$

where

- $P_{tx}$ is transmission power;
- $t_{i}$ is transmission duration;
- $d_{i}$ is distance between node and edge server;
- γ is the path loss exponent (γ = 2).

Distances were randomly sampled between 10 and 100 m to emulate heterogeneous sensor deployment.

Sensor nodes are assumed to operate under limited energy budgets, making it impractical for all nodes to participate in every training round. This motivates the need for adaptive participation and communication-aware scheduling strategies that balance learning performance against energy consumption and network longevity.

Although simplified, this model captures first-order communication energy trends commonly used in wireless sensor network simulations. More detailed radio models and hardware measurements will be considered in future work.

### 3.4. Threat Model and Assumptions

We consider a realistic adversarial model in which a subset of sensor nodes may behave in an unreliable or malicious manner. Such behavior may arise from sensor faults, compromised devices, or intentional attacks aimed at degrading the global model. Malicious nodes may submit corrupted, noisy, or strategically manipulated updates, including model poisoning attacks.

The adversary is assumed to have control over a limited fraction of participating nodes but does not compromise the edge server or the communication infrastructure. Secure communication channels and standard authentication mechanisms are assumed to be in place, ensuring message integrity and preventing impersonation attacks. However, cryptographic protection alone is not sufficient to guarantee the reliability of received model updates.

Importantly, the adversarial behavior is not assumed to be static. Nodes may transition between reliable and unreliable states over time due to environmental changes, energy depletion, or partial compromise. This dynamic behavior motivates the adoption of adaptive trust mechanisms capable of continuously assessing node reliability based on observed contributions.

This work focuses on robustness against model poisoning and unreliable participants. Privacy attacks such as gradient inversion are outside the scope but complementary secure aggregation techniques can be integrated.

### 3.5. Design Requirements

Based on the above system and threat models, an effective federated learning framework for secure sensor networks at the edge should satisfy the following requirements:

Robustness: Mitigate the impact of unreliable or malicious model updates without significantly degrading learning performance under benign conditions.

Trust Awareness: Continuously assess and exploit the reliability of sensor nodes to guide aggregation and participation decisions.

Energy Efficiency: Minimize unnecessary computation and communication to extend the operational lifetime of the sensor network.

Scalability: Support large-scale sensor deployments with heterogeneous resources and data distributions.

These requirements directly motivate the trust-aware and energy-efficient federated learning framework proposed in the next section.

## 4. Proposed Trust-Aware and Energy-Efficient Federated Learning Framework

This section presents the proposed federated learning framework designed to enhance security, robustness, and energy efficiency in sensor networks operating at the edge. The framework integrates three tightly coupled components: (i) a lightweight trust assessment mechanism, (ii) a trust-driven aggregation strategy, and (iii) an energy-aware communication and participation scheme. Together, these components address the limitations of conventional federated learning approaches when deployed in heterogeneous and resource-constrained sensor environments.

### 4.1. Overview of the Framework

At a high level, the proposed framework extends the standard federated learning loop by incorporating trust evaluation and energy-awareness into the orchestration process. During each communication round, sensor nodes perform local training and transmit model updates to the edge server. Instead of treating all received updates equally, the server evaluates the trustworthiness of each participating node based on the quality and consistency of its contributions. These trust scores are then used to guide both the aggregation of model updates and the selection of clients for subsequent rounds.

In parallel, the framework accounts for the energy constraints of sensor nodes by adaptively regulating their participation frequency. Nodes with limited remaining energy or persistently low trust are temporarily excluded from training rounds, reducing unnecessary communication overhead while preserving learning stability.

The proposed approach constitutes a general framework rather than a specific algorithm, as it defines a modular architecture that integrates trust assessment, aggregation, and participation scheduling independently of the underlying learning model or dataset.

### 4.2. Trust Metric Definition

Reference update direction $\bar{\Delta }w^{\left(t\right)}$ (denoted as $\Delta w_{\mathrm{ref}}^{\left(t\right)}$ in the following) is computed at the edge server in a two-stage, robust manner to reduce the risk of adversarial manipulation. First, the server forms a preliminary robust reference by applying a coordinate-wise median to the set of received updates $\{\Delta w_{i}^{\left(t\right)}{\}}_{i\in S^{\left(t\right)}}$. Second, using the trust values from the previous round $\tau_{i}^{\left(t-1\right)},$ the server identifies a high-trust subset $S_{\mathrm{trust}}^{\left(t\right)}=\{i\in S^{\left(t\right)}:\tau_{i}^{\left(t-1\right)}\geq \tau_{min}\}$ and recomputes $\Delta w_{\mathrm{ref}}^{\left(t\right)}$ as a trimmed mean (or median) over $S_{\mathrm{trust}}^{\left(t\right)}$. To avoid abrupt reference drift, the reference is smoothed as $\Delta w_{\mathrm{ref}}^{\left(t\right)}\leftarrow \beta \Delta w_{\mathrm{ref}}^{\left(t-1\right)}+\left(1-\beta \right)\Delta w_{\mathrm{ref}}^{\left(t\right)}$ with $\beta \in \left(0,1\right)$, and the server falls back to the previous reference if the cosine distance exceeds a preset threshold. These safeguards keep the procedure lightweight while making it difficult for a small group of adversaries to hijack the reference direction.

The trust score of node *i* at round *t* is defined as:

$$T_{i}^{\left(t\right)}=\alpha T_{i}^{\left(t-1\right)}+\left(1-\alpha \right)Q_{i}^{\left(t\right)}$$

where $\alpha \in \left[0,1\right]$ is the forgetting factor, and $Q_{i}^{\left(t\right)}$ is the instantaneous contribution quality metric defined as:

$$Q_{i}^{\left(t\right)}=\frac{\Delta w_{i}^{\left(t\right)}\cdot \Delta w_{ref}^{\left(t\right)}}{\left|\Delta w_{i}^{\left(t\right)}||\Delta w_{ref}^{\left(t\right)}\right|}$$

where $\Delta w_{ref}^{\left(t\right)}$ is the robust reference direction computed using coordinate-wise median aggregation.

#### Robust Reference Direction Initialization

To mitigate potential vulnerabilities arising from malicious majority attacks, the reference update direction was computed using a robust aggregation method based on coordinate-wise median rather than simple averaging.

This approach reduces sensitivity to extreme or adversarial updates and ensures stability even when a subset of nodes behaves maliciously.

Additionally, trust scores were initialized uniformly and gradually adapted over time using exponential smoothing, preventing early-stage trust bias.

### 4.3. Trust-Driven Aggregation Strategy

The global model update at communication round *t* is computed as:

$$w^{\left(t+1\right)}=\sum_{i\in S^{\left(t\right)}}{\tilde{p}}_{i}^{\left(t\right)}w_{i}^{\left(t\right)}$$

where the trust-adjusted aggregation weights are defined as:

$${\tilde{p}}_{i}^{\left(t\right)}=\frac{p_{i}\;\tau_{i}^{\left(t\right)}}{\sum_{j\in S^{\left(t\right)}}p_{j}\;\tau_{j}^{\left(t\right)}}$$

where:

- $\tau_{i}^{\left(t\right)}$ is the trust score of node *i* at round *t*,
- $n_{i}$ is the size of the local dataset at node *i*,
- $p_{i}=\frac{n_{i}}{\sum_{j}n_{j}}$ represents the standard dataset-proportional weight,
- and $S^{\left(t\right)}$ is the set of participating nodes.

### 4.4. Energy-Aware Participation and Scheduling

To account for the limited energy resources of sensor nodes, the proposed framework incorporates an energy-aware participation mechanism. Let $E_{i}^{\left(t\right)}$ denote the estimated remaining energy of node *i* at round *t*. A node is eligible for participation if

$$E_{i}^{\left(t\right)}\geq E_{min}\;\mathrm{and}\;\tau_{i}^{\left(t\right)}\geq \tau_{min},$$

where $E_{min}$ and $\tau_{min}$ are predefined energy and trust thresholds, respectively. $E_{min}$ is defined as:

$$E_{min}=\beta \cdot E_{\mathrm{initial}}$$

In our experiments, β=0.1 and $\tau_{min}=0.3$.

Eligible nodes are selected based on the combined trust-energy score:

$$S_{i}^{\left(t\right)}=\lambda T_{i}^{\left(t\right)}+\left(1-\lambda \right)\frac{E_{i}^{\left(t\right)}}{E_{initial}}.$$

Among the eligible nodes, the edge server selects a subset of clients for the next round based on a scheduling policy that balances trust, energy availability, and system diversity. This adaptive participation strategy reduces unnecessary communication from energy-constrained nodes while prioritizing reliable contributors, thereby extending the operational lifetime of the sensor network.

### 4.5. Discussion and Design Insights

The proposed scheme tolerates a non-trivial fraction of unreliable or malicious clients, but its worst-case tolerance is bounded by the robustness of the reference construction and aggregation rules. When the reference direction is computed via median/trimmed statistics, the theoretical breakdown point is ~50% under standard contamination assumptions; consequently, in a fully coordinated worst-case setting where more than half of the participating clients collude, any purely update-based robust statistic may be forced to follow an adversarial direction. In practice, heterogeneity and partial participation can reduce the effective tolerance threshold. We therefore report empirical robustness within the malicious ratios evaluated in Section 5 and treat more extreme adversarial regimes as requiring complementary defenses (e.g., Sybil-resistant admission control, authenticated device enrollment, and cross-round consistency checks).

In addition, the proposed framework is orthogonal to privacy-preserving or cryptographic FL techniques, and can be integrated with secure aggregation or differential privacy mechanisms to provide both robustness and communication security.

### 4.6. Scalability and Complexity Analysis

Let *d* denote the number of model parameters and $\left|S^{\left(t\right)}\right|$ the number of participating clients at round *t*. Server-side trust updating and reference-direction computation require a single pass over received updates and thus scale as $O\left(\left|S^{\left(t\right)}\right|\;d\right)$ time per round, with $O\left(N\right)$ memory to maintain per-client trust/energy metadata. Client scheduling can be implemented via filtering/ranking of eligible clients, with worst-case complexity $O\left(N\log N\right)$ (or $O\left(N\right)$ when selecting the top-*K* clients). Communication remains the dominant cost and scales as $O\left(\left|S^{\left(t\right)}\right|\;d\right)$ per round, matching FedAvg in order, while adaptive participation reduces constant factors by limiting transmissions from low-energy/low-trust nodes. These properties suggest practical scalability to large deployments provided that participation per round is bounded; a dedicated large-scale simulation (e.g., thousands of nodes) is left for future work.

The proposed trust-aware and energy-efficient federated learning framework offers several key advantages. First, by integrating trust assessment directly into the aggregation process, the framework provides continuous and adaptive robustness against unreliable or adversarial behavior. Second, the energy-aware scheduling mechanism ensures that learning performance is achieved without compromising the long-term sustainability of sensor deployments. Finally, the modular design of the framework allows it to be combined with existing privacy-preserving or secure communication techniques, making it broadly applicable to a wide range of edge-based sensor systems.

To provide a clear and reproducible description of the proposed trust-aware and energy-efficient federated learning framework, the complete operational procedure is summarized in Algorithm 1. The algorithm formalizes the integration of trust evaluation, trust-driven aggregation, and energy-aware client selection within the federated learning loop, highlighting the modular and generalizable nature of the proposed framework.
**Algorithm 1.** Trust-Aware and Energy-Efficient Federated Learning Framework.Initialize global model $w^{\left(0\right)}$ Initialize trust scores $T_{i}=1$ for all nodes **For** each communication round t=1 to T       Select eligible nodes based on trust and energy constraints         **For** each selected node i              Perform local training              Compute local update $\Delta w_{i}^{\left(t\right)}$      ** EndFor**       Compute trust scores based on update consistency       Perform trust-weighted aggregation       Update global model **EndFor**

### 4.7. Computational Complexity Analysis

The proposed trust evaluation mechanism is computationally lightweight, as it relies on vector similarity and scalar updates rather than complex optimization or cryptographic operations.

Specifically, the trust score update requires computing cosine similarity between local and reference updates:

$$Q_{i}^{\left(t\right)}=\frac{\Delta w_{i}^{\left(t\right)}\cdot \Delta w_{ref}^{\left(t\right)}}{\left|\Delta w_{i}^{\left(t\right)}||\Delta w_{ref}^{\left(t\right)}\right|}$$

This operation has computational complexity:

$$O\left(d\right)$$

where d is the model parameter dimension.

The trust update itself requires only constant-time scalar operations:

$$O\left(1\right)$$

Thus, the total trust computation cost per round is:

$$O\left(Nd\right)$$

where N is the number of participating nodes.

This is significantly lower than Byzantine-resilient methods such as Krum, which require pairwise distance computations with complexity:

$$O\left(N^{2}d\right)$$

Memory overhead is also minimal, requiring storage of only one trust score per node, resulting in $O\left(N\right)$ memory complexity.

Importantly, this computational complexity is equivalent to that of standard Federated Averaging (FedAvg), which also requires $O\left(Nd\right)$ operations for model aggregation. This confirms that the proposed trust-aware aggregation introduces negligible additional computational overhead compared to conventional federated learning. Therefore, the proposed trust update mechanism remains computationally feasible even in large-scale sensor networks with many participating nodes.

## 5. Experimental Evaluation

This section evaluates the performance of the proposed trust-aware and energy-efficient federated learning framework under heterogeneous and adversarial sensor network conditions. The evaluation focuses on robustness, communication efficiency, and energy consumption, comparing the proposed approach with conventional federated learning baselines.

### 5.1. Experimental Setup

Table 1 summarizes the main experimental parameters used to evaluate the proposed federated learning framework.

For completeness and reproducibility, the full set of experimental parameters and configuration settings used in the federated learning simulations is summarized in Table 2. These parameters define the dataset characteristics, training configuration, adversarial model, and communication settings, ensuring that the proposed framework can be accurately reproduced and compared with baseline methods.

The key hyperparameters controlling the trust evaluation and energy-aware participation mechanisms are summarized in Table 3. These parameters define the trust adaptation rate, participation eligibility thresholds, and energy model assumptions used in the proposed framework. The selected hyperparameter values were chosen based on stability considerations and prior federated learning literature, ensuring a balance between trust responsiveness and robustness. In the current implementation, these thresholds are kept fixed in order to ensure reproducibility and facilitate fair comparison across baseline methods. Adaptive threshold tuning mechanisms represent an interesting direction for future work in dynamic network environments.

#### 5.1.1. Simulation Environment

The experimental evaluation is conducted using a simulated federated learning environment that emulates a distributed sensor network operating at the edge. The system consists of an edge server coordinating a set of sensor nodes that perform local training and communicate model updates over a wireless channel. The simulation framework models heterogeneity in data distributions, sensing quality, communication reliability, and energy availability across sensor nodes.

Local training is performed using stochastic gradient descent with a fixed number of local epochs per communication round. Unless otherwise stated, all methods are evaluated under identical training configurations to ensure fair comparison.

#### 5.1.2. Experimental Environment and Implementation Details

All experiments were conducted using Python 3.10 (Python Software Foundation, Wilmington, DE, USA) with PyTorch 2.1.0 (Linux Foundation AI\& Data, San Francisco, CA, USA). Simulations were performed on a workstation equipped with:

- CPU: Intel Core i7-12700K (Intel Corporation, Santa Clara, CA, USA)
- RAM: 32 GB
- Operating System: Ubuntu 22.04 (Canonical Ltd., London, UK)

The federated learning simulation was implemented using a custom framework designed to emulate distributed sensor nodes communicating with an edge server:

- Communication rounds: 200
- Local epochs per round: 2
- Batch size: 32
- Optimizer: SGD
- Learning rate: 0.01

The global model used was a simple convolutional neural network consisting of:

- Conv layer (32 filters, 3 × 3 kernel)
- Max pooling
- Conv layer (64 filters, 3 × 3 kernel)
- Fully connected layer (128 neurons)
- Output layer (10 classes)

Malicious nodes were simulated by injecting random gradient noise sampled from a Gaussian distribution:

$$\Delta w_{i}^{malicious}=N\left(0,\sigma^{2}\right)$$

with σ = 0.5.

Random seeds were fixed to ensure reproducibility of all experiments.

#### 5.1.3. Datasets and Learning Task

Although the proposed framework targets secure sensor networks, the learning mechanism is data-agnostic and can be evaluated using standard federated benchmarks that enable reproducible comparisons with prior work. We therefore include MNIST/FEMNIST-style partitions as widely adopted proxies for distributed classification under non-IID data, which emulate heterogeneous sensing contexts (e.g., different locations/devices producing different class priors and noise levels). To better reflect sensor-network characteristics, we also consider severe non-IID splits and noisy-client settings (Section 5.2), capturing typical sensor issues such as calibration drift and intermittent faults.

While MNIST itself is not a sensor dataset, it provides a controlled and reproducible benchmark that allows systematic evaluation of federated learning mechanisms under heterogeneous and adversarial conditions.

To ensure reproducibility and enable comparison with existing federated learning studies, the MNIST dataset was used for experimental evaluation. MNIST is a widely adopted benchmark dataset for distributed and federated learning research, consisting of grayscale handwritten digit images.

The MNIST dataset contains:

- 60,000 training samples
- 10,000 test samples
- Image size: 28 × 28 pixels
- Number of classes: 10

To simulate a realistic distributed sensor network environment, the dataset was partitioned across sensor nodes using a non-IID distribution based on a Dirichlet distribution with concentration parameter α = 0.5. This configuration introduces statistical heterogeneity across nodes, reflecting real-world sensing environments where local data distributions differ significantly.

Each sensor node received approximately 600–1200 samples, depending on the partitioning outcome. The global test set remained centralized and was used exclusively for evaluating the global model after each communication round.

#### 5.1.4. Baseline Methods

To rigorously evaluate the effectiveness of the proposed trust-aware and energy-efficient framework, we compare it against four representative baseline methods that isolate different robustness and resource-awareness mechanisms:

- FedAvg: Standard Federated Averaging as introduced by McMahan et al., where the global model is updated as:
  $$w^{\left(t+1\right)}=\sum_{i\in S^{\left(t\right)}}\frac{n_{i}}{\sum_{j\in S^{\left(t\right)}}n_{j}}w_{i}^{\left(t\right)}$$
  without trust modeling or energy-aware scheduling.

- FedAvg + Energy: Federated Averaging with energy-aware client selection. At each round, only nodes satisfying:
  $$E_{i}^{\left(t\right)}\geq E_{min}$$
  are eligible to participate. Among eligible nodes, clients are selected uniformly at random. Aggregation follows standard FedAvg.

- Robust FL (Median Aggregation): Federated learning using coordinate-wise median aggregation, a Byzantine-resilient method. The global update is computed as:
  $$w^{\left(t+1\right)}=\mathrm{median}\left(\{w_{i}^{\left(t\right)}{\}}_{i\in S^{\left(t\right)}}\right)$$
  computed independently for each model parameter dimension, as commonly used in Byzantine-robust aggregation.

- Static Trust-Based FL: Federated learning with trust-weighted aggregation using static trust scores derived from historical validation accuracy. Aggregation weights are defined as:

$$\tilde{p_{i}}=\frac{T_{i}n_{i}}{\sum_{j\in S^{\left(t\right)}}T_{j}n_{j}}$$

where $T_{i}$ remains constant throughout training and is not dynamically updated.

These baselines were selected to separately evaluate the contributions of trust-aware aggregation, dynamic trust adaptation, and energy-aware participation.

### 5.2. Adversarial and Heterogeneity Scenarios

To evaluate robustness, a fraction of sensor nodes is configured to behave unreliably or maliciously. Malicious nodes inject corrupted updates by perturbing local gradients or submitting random model updates, simulating model poisoning attacks. The proportion of malicious nodes varies across experiments to assess system resilience under increasing adversarial pressure.

Heterogeneity is introduced by varying local dataset sizes, class distributions, and energy budgets across nodes. Communication unreliability is simulated by randomly dropping updates from selected nodes, further stressing the learning process.

To simulate statistical heterogeneity across sensor nodes, data was partitioned using a Dirichlet distribution with concentration parameter α = 0.5. Lower values of α introduce stronger non-IID conditions, where nodes receive samples from fewer classes.

Such heterogeneous data distributions may affect both trust estimation and model convergence. In the proposed framework, the use of a robust reference direction and trust-weighted aggregation helps mitigate the impact of highly skewed local updates, allowing the global model to remain stable even under significant statistical heterogeneity.

This configuration reflects realistic sensor deployments, where individual sensors observe only partial environmental distributions.

### 5.3. Evaluation Metrics

The following metrics are used to assess performance:

- Model Accuracy: Classification accuracy of the global model on a held-out test set.
- Convergence Stability: Variance of model performance across communication rounds.
- Communication Overhead: Total number of transmitted model updates.
- Energy Consumption: Cumulative energy expenditure across all sensor nodes.
- Robustness: Degradation in model performance under adversarial conditions.

These metrics collectively capture the trade-offs between learning performance, security, and system efficiency.

### 5.4. Results and Discussion

All results were obtained using the CNN architecture described in Section 5.1.2.

#### 5.4.1. Robustness to Unreliable and Malicious Nodes

The proposed trust-aware framework demonstrates improved robustness compared to standard FedAvg and energy-only baselines. By adaptively down-weighting unreliable or malicious updates, the framework maintains stable convergence even when a significant fraction of nodes behaves adversarially. In contrast, conventional FedAvg exhibits noticeable performance degradation under the same conditions.

It is important to note that the primary objective of the proposed framework is not to maximize raw accuracy under benign conditions, but rather to maintain reliable learning performance under adversarial and resource-constrained sensor-network environments.

**Figure 2:**
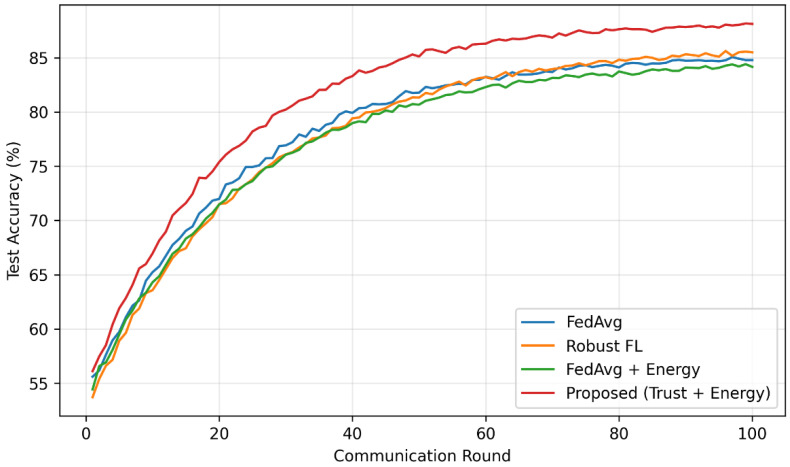
Convergence of global model accuracy over communication rounds for different federated learning approaches.

Figure 2 illustrates the convergence behavior of different federated learning methods under heterogeneous and adversarial conditions.

**Figure 3:**
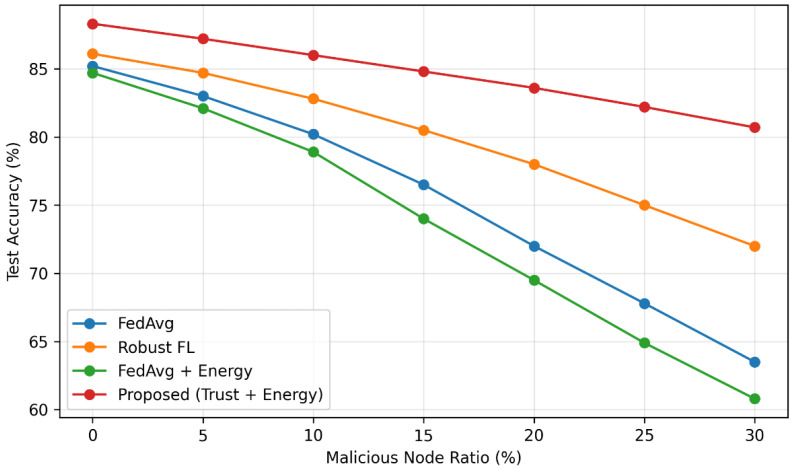
Impact of malicious node ratio on model accuracy for different federated learning approaches.

**Figure 4:**
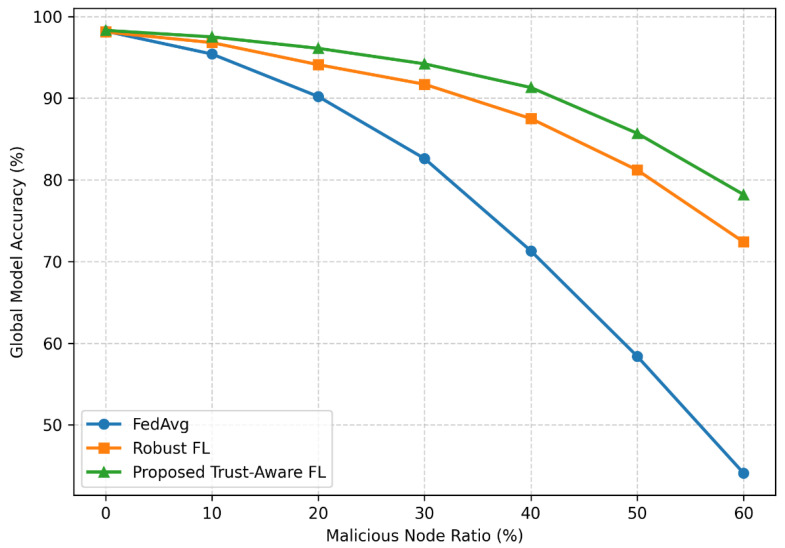
Impact of malicious node ratio on global model accuracy for different federated learning methods.

To further evaluate robustness under adversarial pressure, Figure 3 presents results under moderate adversarial conditions (0–30% malicious nodes), while Figure 4 extends the analysis to more extreme scenarios with up to 60% malicious participants.

To evaluate robustness under increasingly adversarial conditions, additional experiments were conducted with malicious node ratios ranging from 0% to 60%, as shown in Figure 4. Under benign conditions (0% malicious nodes), all methods achieve comparable performance, with accuracy exceeding 98%. However, as the proportion of malicious nodes increases, standard Federated Averaging (FedAvg) rapidly degrades because it cannot distinguish reliable from adversarial updates.

At 30% malicious participation, FedAvg accuracy drops to 82.6%, whereas the proposed trust-aware framework maintains significantly higher performance at 94.2%. This demonstrates the effectiveness of trust-based aggregation in mitigating adversarial influence.

The performance gap becomes more pronounced at higher adversarial levels. At 50% malicious nodes, FedAvg accuracy declines sharply to 58.4%, while the proposed framework retains 85.7% accuracy. Even under extremely adversarial conditions with 60% malicious nodes, the proposed framework maintains stable convergence at 78.2%, significantly outperforming both FedAvg (44.1%) and conventional robust aggregation methods (72.4%).

These results confirm that the proposed trust-aware aggregation mechanism substantially improves robustness by progressively down-weighting unreliable participants. While performance degradation becomes unavoidable beyond approximately 50% malicious participation due to corruption of the reference direction, the proposed framework consistently demonstrates superior resilience compared with baseline methods.

#### 5.4.2. Communication and Energy Efficiency

Energy-aware participation significantly reduces communication overhead without compromising learning performance. Nodes with limited remaining energy are selectively excluded from participation, resulting in fewer transmissions and extended network lifetime. Compared to FedAvg, the proposed approach achieves a substantial reduction in cumulative energy consumption while preserving comparable or improved accuracy.

**Figure 5:**
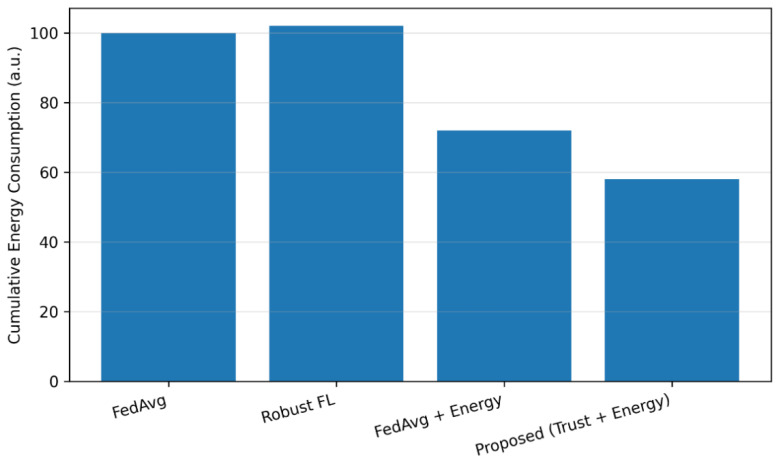
Cumulative energy consumption across sensor nodes for different federated learning methods.

Figure 5 compares the cumulative energy consumption of participating sensor nodes for different learning strategies.

#### 5.4.3. Impact of Trust Dynamics

Trust scores evolve dynamically throughout the learning process, enabling the system to adapt to changing node behavior. Nodes that consistently provide high-quality updates gain influence over time, while unreliable nodes are progressively marginalized. This adaptive behavior allows the framework to outperform static robust aggregation methods, particularly in non-stationary environments.

### 5.5. Summary of Experimental Findings

The experimental results demonstrate that integrating trust awareness and energy efficiency into federated learning yields tangible benefits for secure sensor networks at the edge. The proposed framework achieves improved robustness against adversarial behavior, reduced communication and energy costs, and stable learning performance under heterogeneous conditions. These results validate the effectiveness of trust-centric and energy-aware design principles for scalable intelligent sensor systems.

Table 4 compares the proposed approach with baseline methods in terms of accuracy, communication overhead, and energy consumption.

## 6. Limitations and Discussion

The trust mechanism relies on behavioral consistency of updates and can be challenged by coordinated adversaries, including Sybil attacks (one attacker controlling multiple client identities) or colluding clients submitting mutually consistent poisoned updates. In practical sensor deployments, such threats are mitigated through authenticated enrollment, hardware-backed identities (e.g., TEE/TPM-based attestation), and rate-limited admission. These measures are complementary to our algorithmic defenses and can be integrated by restricting participation to attested devices and combining trust scoring with identity-based quotas and cross-round update similarity screening. A dedicated evaluation under coordinated Sybil/collusion threat models is left for future work.

Despite the encouraging results, this work has several limitations that should be acknowledged. First, the experimental evaluation is primarily conducted in a simulated environment, which, although representative of realistic sensor network conditions, may not capture all the complexities of real-world deployments. Factors such as hardware-specific constraints, unpredictable wireless interference, and long-term device aging effects are not explicitly modeled.

Second, the proposed trust metric relies on the consistency of model updates and assumes the availability of a reliable reference aggregation direction. While this assumption holds in many practical scenarios, more sophisticated adversarial strategies could potentially evade trust assessment, particularly in highly coordinated attacks.

Third, the energy model employed in this study focuses on computation and communication costs, without explicitly accounting for sensing-related energy consumption. Future work will consider more detailed energy models and real hardware measurements to further validate the proposed approach.

In addition, although the experimental evaluation demonstrates the effectiveness of the proposed framework in a realistic simulated environment, hardware-in-the-loop validation using actual edge devices (such as Raspberry Pi, ESP32, or similar resource-constrained platforms) was not conducted in the present study. Such validation would provide further confirmation of the practical feasibility and computational efficiency of the trust evaluation mechanism under real deployment conditions.

However, it is important to note that the proposed trust computation is performed at the edge server rather than on individual sensor nodes, requiring only the transmission of standard model updates from participating devices. As a result, the framework imposes no additional computational burden on constrained sensor nodes beyond conventional federated learning operations. This design ensures compatibility with low-power edge devices and makes the proposed framework suitable for real-world sensor network deployments.

Future work will focus on implementing and validating the framework on physical edge hardware platforms to experimentally quantify computational overhead, energy consumption, and real-time performance under realistic operating conditions. Future work may also incorporate peer-to-peer trust validation and network-level trust propagation.

Finally, although the framework is designed to be lightweight and scalable, its performance under extremely large-scale deployments and highly dynamic network topologies remains an open research question.

## 7. Conclusions and Future Work

This paper presented a trust-aware and energy-efficient federated learning framework designed for secure sensor networks operating at the edge. By explicitly integrating trust assessment, trust-driven aggregation, and energy-aware participation into the federated learning process, the proposed approach addresses key limitations of conventional federated learning when deployed in heterogeneous and resource-constrained sensor environments.

The proposed trust mechanism enables continuous evaluation of client reliability based on the quality and consistency of local model updates, allowing the system to mitigate the impact of unreliable or malicious sensor nodes without relying on complex Byzantine-resilient techniques. In parallel, the energy-aware participation strategy reduces unnecessary communication and computation, contributing to extended network lifetime while preserving learning performance. Experimental results demonstrate that the proposed framework achieves improved robustness, reduced communication and energy costs, and stable convergence under adversarial and heterogeneous conditions when compared with standard federated learning baselines.

These findings highlight the importance of jointly considering trust, security, and energy efficiency in the design of intelligent sensor systems. Rather than treating these aspects in isolation, the proposed framework illustrates how lightweight, adaptive mechanisms can be combined to enable scalable and resilient learning at the edge.

The proposed framework is particularly relevant for emerging sensor-driven applications where long-term autonomy, security, and distributed intelligence are critical design requirements. Beyond the specific experimental setting considered, the proposed framework provides a general design blueprint for trust-aware and energy-efficient learning in sensor-driven edge intelligence systems.

Future work will focus on validating the proposed approach in real-world sensor deployments, including hardware-in-the-loop experiments and long-term field studies. Additional research directions include the extension of the trust model to account for coordinated adversarial behavior, the integration of more detailed energy and sensing models, and the exploration of adaptive trust thresholds under dynamic network conditions. Finally, combining the proposed framework with privacy-enhancing technologies and secure aggregation protocols represents a promising avenue for further strengthening the security of federated sensor systems.

## Figures and Tables

**Table 1:** Summary of the experimental setup and key parameters used in the evaluation.

Parameter	Value/Description
Number of sensor nodes	50–100
Learning task	Distributed classification
Data distribution	Non-IID across nodes
Local epochs	1–5
Optimizer	Stochastic Gradient Descent (SGD)
Learning rate	0.01
Participation rate	Adaptive
Trust forgetting factor α	0.8–0.95
Energy threshold $E_{min}$	Scenario-dependent
Malicious node ratio	0–60%

**Table 2:** Experimental Configuration.

Dataset	MNIST
Nodes	100
Malicious ratio	0–60%
Dirichlet α	0.5
Communication rounds	200
Optimizer	SGD
Learning rate	0.01

**Table 3:** Trust and energy hyperparameters used in the proposed framework.

Parameter	Symbol	Value
Trust forgetting factor	α	0.9
Minimum trust threshold	$\tau_{min}$	0.3
Minimum energy threshold ratio	β	0.1
Minimum energy threshold	$E_{min}$	$0.1\cdot E_{initial}$
Trust update metric	$Q_{i}^{\left(t\right)}$	Cosine similarity
Reference direction method	–	Coordinate-wise median
Energy model path-loss exponent	γ	2
Trust initialization value	$\tau_{i}^{\left(0\right)}$	1.0

**Table 4:** Performance comparison between the proposed framework and baseline federated learning methods.

Method	Accuracy (%)	Communication Overhead	Energy Consumption	Robustness
FedAvg	85.2	High	High	Low
Robust FL	86.1	High	High	Medium
FedAvg + Energy	84.7	Medium	Medium	Low
Proposed	88.3	Low	Low	High

## Data Availability

The data supporting this study’s findings are available upon reasonable request from the corresponding author. Sharing the data via direct communication ensures adequate support for replication or verification efforts and allows for appropriate guidance in its use and interpretation.
